# An Update from the Benchmark Survey of phactMI™ Member Companies on Providing Medical Information in the Digital Space

**DOI:** 10.1007/s43441-023-00587-1

**Published:** 2023-11-10

**Authors:** Sheena Dupuy, Laura Opincar, Kirtida Pandya, Susan Wnorowski, Marianne Cuellar, Jennifer Riggins, Evelyn R. Hermes-DeSantis

**Affiliations:** 1 AstraZeneca Pharmaceuticals, One MedImmune Way (Building 200), Gaithersburg, MD 20878 USA; 2 Global Medical Information & Communication, AstraZeneca Pharmaceuticals, One MedImmune Way (Building 200), Gaithersburg, MD 20878 USA; 3 Medical Affairs, Sandoz, Inc., 100 College Rd. West, Princeton, NJ 08540 USA; 4 US Medical Information and Patient Safety, Ipsen Biopharmaceuticals, Inc, Cambridge, USA; 5 Ipsen Biopharmaceuticals Canada Inc., 5050 Satellite Drive, Suite 500, Mississauga, ON L4W 0G1 Canada; 6 phactMI, Glen Mills, PA USA; 7 phactMI, 142 Glen Mills Rd., PO Box 23, Glen Mills, PA 19342 USA

**Keywords:** Medical Information, Websites, Technology, Benchmarking, Pharmaceutical industry, Survey, phactMI

## Abstract

**Objective:**

The Pharma Collaboration for Transparent Medical Information (phactMI™) benchmarking survey of 32 pharmaceutical companies describes the use of technology by Medical Information Departments.

**Methods:**

A survey was distributed to phactMI™ member companies in June 2022 and included 79 closed and open-ended questions. The survey’s six sections included demographics, chatbot, social media, instant messaging applications, websites, and technology.

**Results:**

Most Medical Information Departments have implemented innovative technology since 2019 with the main driver of remaining up-to-date. A total of 94% have a Medical Information website. Of those with a Medical Information website for healthcare professionals (HCPs), 97% allow for self-authentication. Most HCP-based websites have webforms for inquiries and 1–800 numbers, while only few offer video chat, chatbot, and the ability to identify local representatives. These websites also link to clinicaltrials.gov, publications, posters, and congress materials. Only 30% have a website for patients/caregivers. Most websites are discoverable by Google™. Awareness of Medical Information Websites occurs in a variety of ways, with most using multiple strategies to reach HCPs. There is wide variation in the technology platform used for the core functions of Medical Information.

**Conclusion:**

As technology continues to advance and omnichannel content remains a key objective, Medical Information needs to remain agile and transformative in their strategic and tactical planning and execution. Based on this benchmarking survey, the authors recommend that Medical Information Departments focus on expanding services for patients/caregivers, leverage digital innovations, expanding awareness, building efficiencies in workflow through technology, and continually improving website functionalities with innovative technologies.

**Supplementary Information:**

The online version contains supplementary material available at 10.1007/s43441-023-00587-1.

## Introduction

Digital innovations have transformed communications and daily operations of almost every business and profession. The rate at which such innovations have evolved and been adopted has accelerated significantly due to the Coronavirus Disease 19 (COVID-19) pandemic which necessitated a rapid pivot to maintain engagement in a virtual world [1]. These advancements have impacted how many industries operate, including in the Medical Information sectors of pharmaceutical companies, which serve as the hub of materials for healthcare professionals (HCPs), patients, and caregivers [2]. No studies to date have assessed how technological advancements since the onset of the COVID-19 pandemic have influenced how Medical Information Departments make medical materials available.

In 2018, Pharma Collaboration for Transparent Medical Information (phactMI™) conducted a relevant benchmarking survey which explored how 27 biopharmaceutical companies in the US are providing medical information in the digital space. [3] The results of the survey demonstrated the need for additional services for patients, innovative formats of delivery of information, additional website functionalities, improvement in search engine optimization (SEO), and innovative presentation of information. The Medical Information Leaders in Europe (MILE) published a framework for the digital provision of Medical Information. The four guiding principles of the framework are an optimal user experience, HCP authentication, surfacing scientific content, and content [4]. In forward looking publications, the utilization of digital to maximize the impact of Medical Affairs and Medical Information and provide an omnichannel, customer-centric experience is a focus [5, 6]. A lynchpin in that experience within Medical Information is through a website and other digital channels. In embracing an omnichannel approach to providing medical information—when, where, and how HCPs want or need the information—Medical Information websites will continue to be a prominent channel. However, there are variabilities in content and format offerings, search engine optimization, search features, and access across the industry.

PhactMI™, a non-profit consortium of Medical Information leaders of the pharmaceutical industry, was created in part to address the issues of HCPs’ awareness and access of Medical Information services in the pharmaceutical industry. This technology benchmark survey serves as a follow-up to the 2018 survey with the objectives of assessing the current landscape of technology, changes in the usage of digital innovations, as well as how technology can assist pharmaceutical companies to better serve HCPs, patients, and caregivers.

## Materials and Methods

In this analysis, 32 US pharmaceutical member companies of phactMI™ voluntarily participated in an electronic survey that was launched in June 2022. The survey included 79 (closed and open-ended) questions with categories including: demographics, chatbot, social media, instant messaging applications (apps), different features and functionality of websites, and general questions on technology platform solutions. The survey was open for 60 days. Responses were automatically saved so that the participant could return to complete the survey at any time during this period. Information was de-identified and analyzed in aggregate.

The survey was set up using skip logic design, in order to eliminate questions not relevant given earlier responses. The survey began with questions concerning company demographics based on January – December 2021 including the number of products supported, number of product launches in the last 12 months, number of different channels for communicating medical information, updates on innovative technologies launched since 2019, and any impact of these technologies.

The next section collected data on the use of social media and instant messaging applications. Questions were tailored to companies who responded that they implemented the specific technologies. Data collected included the types of technology used, information provided, and utilization.

The third section focused on Medical Information websites. Questions in this section focused on maintenance, access, type of user-based site (i.e., HCP vs. Patient), authentication methods, functionality including chatbot, and content/format availability. Additional areas explored included customer satisfaction surveys, website discoverability and search engine optimization (SEO), search functionality on the website, and metrics.

The last section of the survey evaluated how to broaden the reach of these technologies to the end users, technology use in the overall content workflow, and to learn of other technology enhancements currently on the horizon. (Supplementary material for full survey).

## Results

### Demographics

All 32 (100%) companies responded to the survey. There was a broad distribution of companies by size with 25% (8/32) having Medical Information responsibilities for less than 5 products, 28% (9/32) having between 6 and 25 products, 16% (5/32) having between 26 and 50 products, and 31% (10/32) having greater than 50 products. A majority (20/32, 63%) of companies had at least one product approved in the last 12 months, accounting for 68 new products on the market.

While 38% (12/32) of the Medical Information groups are responsible for US only functions, 25% (8/32) are Global only and the remaining 38% (12/32) have both US and Global responsibilities. The core responsibility of Medical Information is responding to unsolicited requests for information on their products from HCPs or patients. While there are many channels for receiving inquiries, 63% of inquiries were received through the Medical Information Call Centers, followed by 10% from the website, 2% from chatbot, 1% from a medical information app, and 24% from other channels. These other channels included field medical, sales representatives, medical booth at congresses, mail or e-mail, and Customer Relationship Management (CRM) software.

**Figure. 1:**
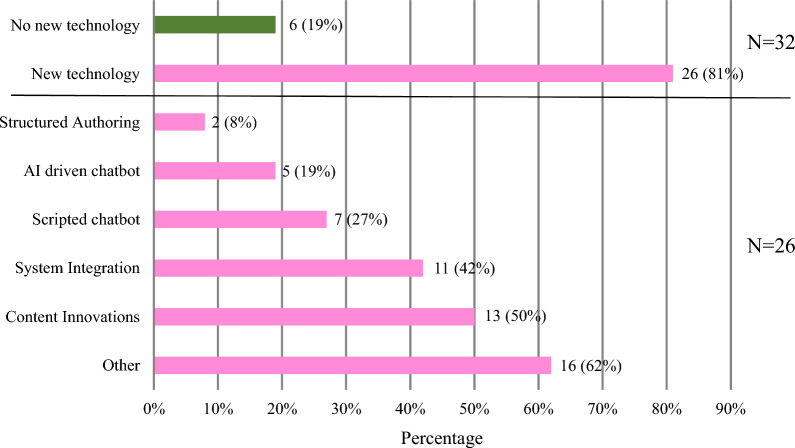
New technologies implemented since 2019.

A majority, 81% (26/32) of Medical Information Departments have implemented innovative technology since 2019. (Fig. 1) Content innovations included infographics, health literate style of writing, conversion to HTML format, shorter documents, incorporation of graphics, next generation Scientific Response Documents (SRD), and patient summaries. Other technology included virtual Medical Information booth, new Medical Information database, Medical Information analytics, video or live chat, data visualization tools for metrics, and a temperature stability calculator. The main driver for the implementation of the technology was to remain up to date with industry changes (24/26, 92%), followed by customer expectations (17/26, 65%), omnichannel engagement strategy (14/26, 54%), internal drivers (11/26, 42%), and finally COVID-19 (6/26, 23%). A majority of companies that implemented new technology, 54% (14/ 26), felt that technology did not impact personnel resources, while 31% (8/26) felt that there was an increase in workload or they needed additional personnel. Table 1 delineates some of the specific comments concerning the impact from technology.

**Table 1.** Technology impact on resources (some specific comments)

Impact	Comments
Increase in resources	We have an Information Technology (IT) operation individual now to help with maintaining our databases
	More personnel needed for technology development and new and existing content management and migration
	Additional training needs. Continued adoption of hybrid employee/vendor model
	Seen an increase in need for additional resources to manage additional channels
	Increase in IT resources and collaboration with Global Medical Information (GMI); more of the GMI's team time spent on technology enhancements–websites, systems, analytics
	Increased use of authoring vendor
Initial increase	The first iterations have resulted in increased task time for some agents (eg, frontline telephone colleagues) but continuous improvements are seeing normalization and expected further improvement. Tasks and features for authoring of content are better than in previous systems and data collection is improving. Addition of resources to support technology enhancements are needed
Improvement	Improved workflows and organization of letter priority have been improved with additional software applications
No impact	Volume not enough to change resource allocations
	Technology implementation has not driven a direct change to staffing personnel currently
	Leveraged technology to streamline insight reporting and foster transparency within the company. There are no changes in our staffing model due to technology utilization
	Personnel resources have been largely unaffected due to technological changes
	Not getting new head count so optimizing current team dynamics by taking advantage of technology/solutions
	Implementation of Chatbot has allowed for approximately 36% of our inquiries to be addressed in an automated fashion. Also, the downloading of congress posters from the Medical Resources Website has reduced the volume of inquiries necessitating manual processing. The impact of the implemented technology has allowed us to stay flat in terms of staffing, despite the significant increase in inquiries

### Social Media

**Figure. 2:**
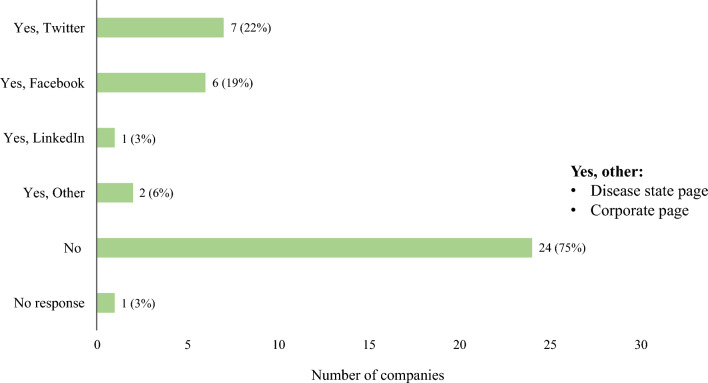
Responding to unsolicited requests through corporate social media page (*n* = 32).

Company Medical Information personnel are becoming more involved in answering unsolicited requests on social media with 22% (7/32) following processes to answer requests on Twitter®, 19% (6/32) on Facebook®, and 3% (1/32) on LinkedIn®. These percentages have increased from 2018 for Twitter® and Facebook® (4%, 1/27, for each) and have remained consistent for LinkedIn® at 4% (1/27). (Fig. 2) Additionally, 3 companies (9%) reported having their own Medical Information Twitter page (2 with < 1 K, 1 with > 5 K followers), 2 companies (6%) reported having a Medical Information LinkedIn® page (1 > 5 K, 1 < 1 K followers) and 1 company (3%) reported having a Medical Information-specific Facebook® page with > 5 K followers. On Medical Information-specific social media pages, a large variation in posts per week existed, ranging from 0–7 posts per week for Twitter® and LinkedIn® and 3–5 posts per week for Facebook®.

### Instant Messaging

Instant Messaging apps allow for texting or chat within a specific application and are not part of a website. Instant Messaging apps are being used regionally by 13% (4/32) of companies: 2 companies use WeChat (China), 1 uses WhatsApp (Brazil, Mexico), and 1 is using ChatNow. Instant Messages are being serviced by in-house personnel (2/4; 50%) and third-party contact centers (2/4; 50%).

### Website

A total of 30/32 (94%) companies responded they provide medical information through a company Medical Information website in comparison to 74% (20/27) in 2018. Eight companies (27%, 8/30) use an existing commercial platform to house their Medical Information website including Salesforce (2/30), Amazon Web Services (1/30), Drupal™ (1/30), SciMax (1/30), MMI (1/30), Toolehouse (1/30) and Amazon experience manager (1/30). Twenty-one companies (70%) have a custom Medical Information website developed and one company (3%) did not respond to this question. Web content management systems varied across companies: Adobe® Experience Manager (9/30), Drupal™ (6/30), WordPress® (3/30) and other (12/30; including Veeva, IRMS CM, Salesforce, Advenio, and custom developed systems). In 2018 no one used Adobe Experience Manager, Veeva, or WordPress®. Salesforce (3/27) and other (8/27) were the most popular options in 2018. Drupal™ was used by 2/27 companies in 2018 vs. 6/30 in 2022. Medical Information websites are maintained internally (16/30), externally through a vendor (9/30) or both (5/30). If maintained internally, departments responsible for this activity include Medical Affairs/Medical Information and/or Business Technology/Information Technology. Vendors responsible for maintenance include Docmation, PRECISION, Cognizant, FFW, InTouch and Anju. Overall, 50% (15/30) do provide a link to their Medical Information website from their branded sites, with 7/30 providing links on all their branded websites and 8/30 providing links on some of their branded websites. Only two companies provide a link from their branded website(s) to their patient website (1 for all products, 1 for some products). When asked whether Medical Information websites are part of broader Medical Affairs websites, 11/30 companies responded yes, 18/30 responded no and 1/30 did not respond.

### HCP Website

Of the 30 respondents with a Medical Information website, 29 are designed for HCPs. All of those site (29/29, 100%) allow for self-authentication. One company responded that they allow for self-authentication, validation (user’s name and National Provider Identifier (NPI) number or other identifying information), and full registration (multiple pieces of information). For non-US based websites, nine allow for self-authentication, seven require validation, and five require full registration. Two companies (7%) have US websites with registration to view specific content and three companies have non-US websites with this same feature. Self-authentication rates increased from 41% (11/27) in 2018 to 100% in 2022.

Regarding available functionalities on HCP websites, a majority (27/29, 93%) of companies have webforms to submit medical information inquiries and 1–800 numbers. Few companies offer video chat (2/29, 7%), chatbot (7/29, 24%) and the ability to identify local representatives (6/29, 21%). Chatbot usage increased to 24% in 2022 from 4% in 2018.

Twenty-five percent (8/32) of survey respondents using various chatbot vendors (Internal-2, Lifelink-1, Indegene®-1, IBM® Watson-1, ConversationHEALTH-3) have implemented a chatbot to help provide medical information services at their company. This compares to only 1 company in 2018. Of these eight companies, the majority (7/8) have a button-based component to their chatbot of which 38% (3/8) have a free text Artificial Intelligence (AI) component in addition to button options, and 13% (1/8) of chatbots have voice driven capabilities. All (3/3) companies with free text/AI capabilities feel that the Natural Language Processing (NLP)/AI interprets the free text and gets the HCP the correct information. Chatbots provide many types of information including SRDs (6/8; 75%), short response Q&A (5/8; 63%), links to websites (5/8; 63%), product information (PI) language or links to PI (5/8; 63%), technical information only (2/8; 25%), and links to publications (1/8; 13%). Many companies (3/8; 37.5%) have been able to free-up or redistribute up to 20–35% of contact center agent time through their chatbot implementation. Noted limitations of chatbots include training and making late breaking data available on Day 0 and only covering the top questions received, not all potential questions. Of those companies without a chatbot (*n* = 24), 13 are considering implementing and 9 are not considering implementing due to cost, regulatory, legal, and compliance concerns, as well as the oversight needed, and time and technology concerns.

**Figure. 3:**
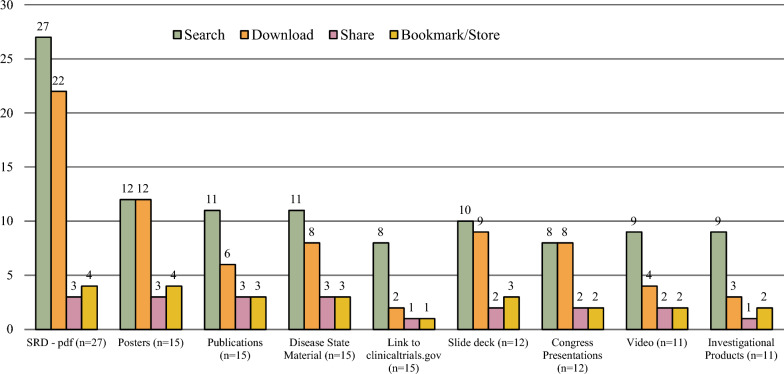
Access and storage of information from Medical Information website (*n* = 29*). *Of the remaining 3 companies, 2 do not have a website and 1 does not have an HCP website.

**Figure. 4:**
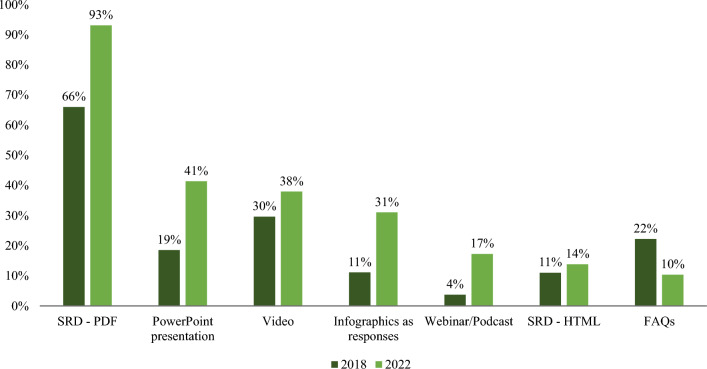
Availability of information/content on Medical Information websites 2018 (*n* = 27) vs. 2022 (*n* = 29). HTML = Hypertext markup language, FAQ = frequently asked questions.

Eleven companies (11/29, 38%) answered they offer other functionalities on the HCP website including: a temperature stability calculator, disease state education, list of key journal publications, Medical Information appointment scheduling, search retrieval of SRDs, clinical trial inquiry submission and links to clinical trials and investigator sponsored research. Various content types are available on HCP website to search, download, share, and bookmark. (Fig. 3) In comparison to 2018, there is an increase in the availability of SRDs, PowerPoint presentations, videos, infographics, and webinars/podcasts. The only content that showed a decrease was Frequently Asked Questions (FAQs) which declined from 22% in 2018 to 10% in 2022 (Fig. 4). As of 2022, Medical Information websites are now offering access to clinicaltrials.gov (52%), publications (52%), posters (52%), and congress materials (41%). Six companies identified as having HTML formatted SRDs; the content was developed by an outside agency (2/6, 33%), internally developed (3/6, 50%), or through an automated process (1/6, 17%).

### Patient Websites

Of the 30 respondents having a Medical Information website, 9/30 (30%) have a website for patients/caregivers. Functionalities included in these patient websites include a 1–800 number (8/9, 89%), webform to submit unsolicited requests (6/9, 66%), webform to submit adverse events/product complaints (AE/PC) (4/9, 44%), and chatbot (2/9, 22%). Information available on the patient website includes SRDs in PDF format (5/9, 56%), links to clinicaltrials.gov (4/9, 44%), interactive tools (2/9, 22%), infographics (1/9, 11%), medically (1/9, 11%) and commercially (1/9, 11%) created patient education materials. Three companies (3/9, 33%) answered that they provide patient information leaflets and patient financial support.

### Search Functionality Within Medical Information Websites

**Figure. 5:**
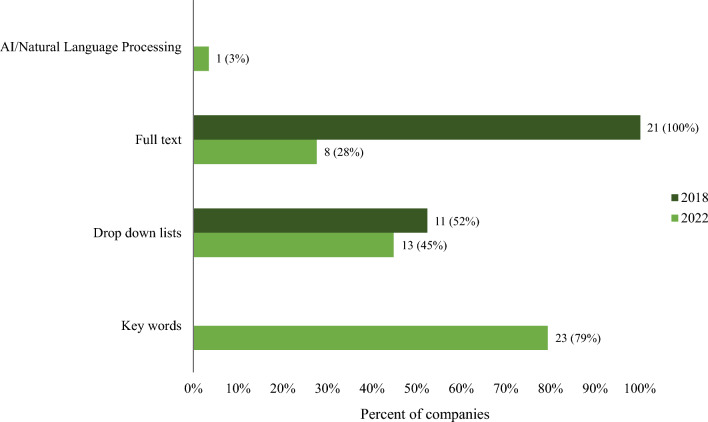
Search functionality on Medical Information Websites – 2018 (*n* = 21) vs. 2022 (*n* = 29). AI = artificial intelligence.

All of the Medical Information websites for HCPs featured a search function (29/29, 100%). Interestingly, four sites (4/9 44%) also provide a search function on their Medical Information website for patients. Search functionality (respondents could select more than 1 option) included key words (23 sites), drop-down lists (13 sites), full-text search (8 sites), and AI/NLP (1 site). (Fig. 5) Most sites (16/26 respondents, 62%) impose a limit on the number of search results returned (1–3 results, 2; 4–5 results, 6; 6–10 results, 4; > 10 results, 4). Ten sites (10/26, 38%) had no limits. The rationales for sites with no limits included: since there are limited responses posted on the website a limit to the search is not needed; the use of key words negates the need for a limit; a limitation of system functionality; and the key word tag imposes automatic limits. One company noted that while they did not impose a limit on search results, the key word tags impose a limit; however, this means of search is not user-friendly and will be changing in the future. The formats of responses returned in search results included SRDs in PDF (26/28, 93%) and HTML (6/26, 21%) formats, slide decks (8/28, 29%), publications (6/28, 21%), posters (9/28, 32%), congress materials (6/28, 21%), and FAQs (3/28, 11%).

### Discoverability of Medical Information Websites

**Figure. 6:**
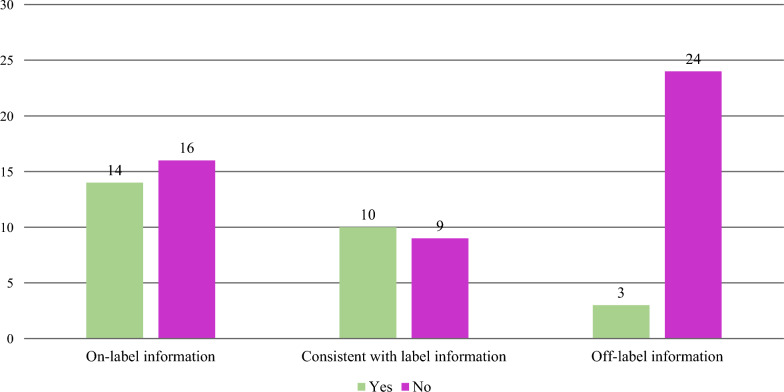
Discoverability of content on Google™ or other search engines (*n* = 30). Note Not all companies responded yes/no to the discoverability of consistent with label or off-label information.

Of the 30 respondents having a Medical Information website, 29/30 websites (97%) are discoverable by Google™ and/or other search engines. This is an increase from 9/25 (36%) sites in the 2018 survey. On-label information is directly discoverable within the search results from these websites in 14 companies (47%), consistent-with-label information in 10 companies (33%), and off-label information in three companies (10%). (Fig. 6) Search engine optimization (SEO) resources were allocated in five companies (5/30, 17%) and included both internal and external/vendor groups. Additionally, four companies (4/30, 13%) indicated using search engine marketing (paid search) to facilitate customers finding their content.

### Customer Satisfaction Surveys

A customer satisfaction survey is conducted by less than 50% of the companies (14/30, 47%). The most common time for the survey to be served up is at the end of the interaction (7/14, 50%); other avenues include a static link on page (2), a link in footer (2), available to selected individuals (1), and in the “Contact us” section of an SRD (1). Regardless of the method of survey provision, the response rate from both patients and HCPs is < 1%. A variety of question formats are used in the customer satisfaction survey such as a Likert scale, multiple choice, yes/no, and open-ended questions. These questions explore the customers’ overall experience, content quality and level of information, value of information to support patient care or clinical decision, customer effort, or format satisfaction.

### Metrics

**Figure. 7:**
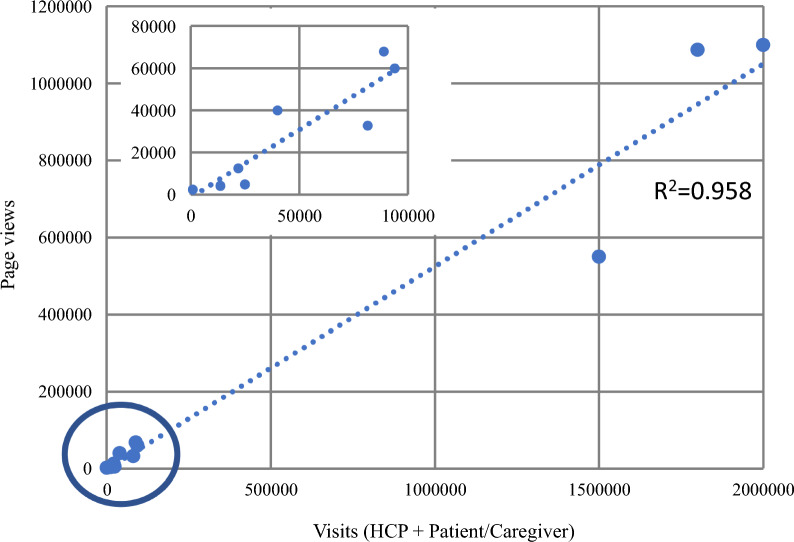
Number of visitors vs. Number of pages viewed.

Overall, 88% (28/30) of the companies collect website metrics. Of the 25 who responded as to tools used for tracking website metrics, most of them use Google™ Analytics (13/25, 52%) or Adobe® Analytics (7/25, 28%). The capture rate for key metrics analyzed include traffic on the website in terms of visitors per year (16/28, 57%), page views annually (12/28, 43%), frequency of same visitor (11/28, 39%), volume of content download (8/28, 29%), and average time spent on the website (10/28, 36%). There was an association between the number of visitors and the number of pages viewed. (Fig. 7) The route by which customers access the website included through Google™ [27%] or the corporate [14%] or brand [12%] site.

### Medical Information Website Awareness

**Figure. 8:**
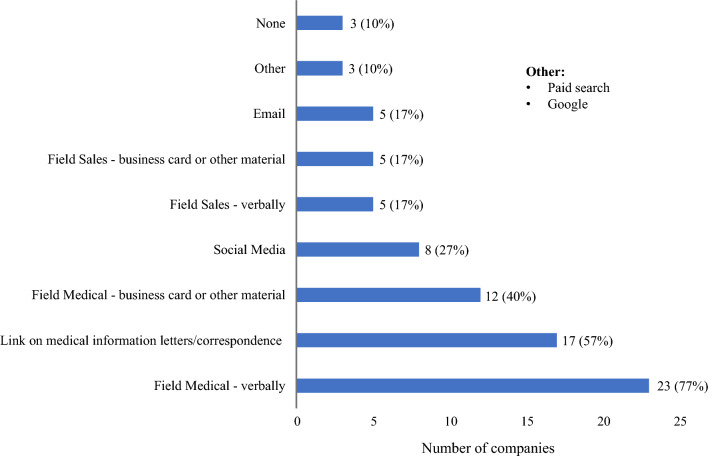
Strategies used to inform HCPs of the Medical Information website (*n* = 30).

Companies increase awareness of their Medical Information Websites in a variety of ways, with most using multiple strategies to reach HCPs. The most common strategy, 77% (23/30), is relying on field medical to verbally inform HCPs of the existence of the Medical Information website. This was followed by 60% (18/30) having a link on Medical Information letters and/or correspondences, and 40% (12/30) having a business card or other material left behind by field medical. (Fig. 8) A total of 53% (10/19) are utilizing social media to broaden the reach of their Medical Information website.

### Medical Information Trends and Insights

Medical Information Departments generate useful and impactful insights and trends based on their interactions with HCPs and patients. The most common method of communicating Medical Information trends and insights to internal stakeholders is via in person or virtual meetings (77%, 24/ 32) followed by e-mail (65%, 20/32) and newsletters (23%, 7/32). There are an array of technology and systems that are used for insights and analytics of inquiries. The most common is Tableau® (36%, 10/28) followed by Qlik Sense® (32%, 9/28). Since 2018, there has been a shift in the use of these two programs with usage of Tableau® increasing from 24% (6/25) and Qlik Sense® (formerly QlikView®) increasing from 4% (1/25). The Medical Information staff is responsible for performing the analytics on inquiry data (68%, 21/31).

Microsoft® Word tops the list of tools being used for content creation (78%, 25/32), followed by Veeva (59%, 19/32) and PowerPoint (34%, 11/32). There is wide variation in the technology platform used for delivering four key functions of Medical Information: customer relationship management (CRM) for inquiries (not Medical Science Liaison (MSL) system), content storage, content workflow management, and fulfillment/package creation. Most companies are utilizing commercial, off-the-shelf platforms compared to home-grown, customized solutions. On average companies utilize two technology platforms (range 1 – 4) to complete these four functions, with some utilizing as many as three platforms for the same function.

**Figure. 9:**
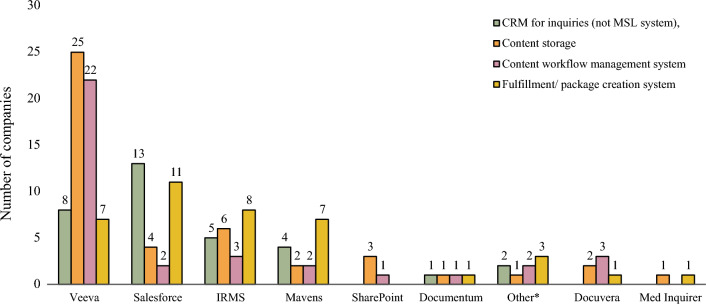
Technology platforms for customer relationship management (CRM) for inquires, content storage, workflow management, and fulfillment/package creation. (*n* = 31). Other include CARA (one GMI), Drawloop, and doDOC.

Veeva, by far, is the most common technology platform used for these various functions, utilized by 87% of respondents (27/ 31). The most common technology used for CRM for inquiries and fulfillment/package creation is Salesforce, and for content storage and content workflow management is Veeva. Other technology platforms used to a lesser degree include IRMS, Mavens, SharePoint, Documentum™, Docuvera™, and MedInquirer (now SciMax MI). (Fig. 9). Only two companies used one platform to accomplish all four tasks, one is using IRMS and one is using CARA from Generis. Most of the technology platforms for the functions of content creation, content workflow management, website framework, and inquiry intake are global in nature. Social media and chatbots tend to be used at the local level.

There are numerous other technologies that Medical Information Departments are working on. (Table 2) The three-year horizon for Medical Information related technology focuses on expansion of websites and chatbots; increase accessibility, access, and personalization of information; innovative formats including infographics and interactive SRDs; structured authoring; and AI and machine learning searches. (Table 3).

**Table 2.** Other technologies

Other technologies currently being worked on
Workflow technology platforms
Exploring changing Medical Information System of Record
Use of new system for inquiry documentation, content storage, and fulfillment
New system for CRM
Novel ways to assemble content
Automating fulfillment process for responses
Website
Web based responses
HCP Portal with On-Demand Library
Improved search functions
Enhancing HCP website (2)
Data analytic tool for website and inquiry tracking
Patient website
Response format
Infographics (3)
Collapsible and expandable views
Channels
Podcasts
Live chat (4)
Chatbot (2)
AI Technology
Verbal AI IVRS
AI technology
Text to speech/speech to text (2)
Automated translation
NLP
AI/ML search
Automation
AI for insights generation, dynamic congress applications
Other technology
Component authoring (2)
Social listening
Digital publishing

Multiple mentions indicated in brackets

*CRM* customer relationship management, *HCP* healthcare professional, *AI* artificial intelligence, *IVRS* interactive voice response system, *NLP* natural language processing, *ML* machine learning

**Table 3.** Future plans for technology

Future technology
Workflow technology platforms
Next generation content management
IRMS integration with Veeva Vault and CRM
Website
Expansion of website
Developing an HCP portal to build a database of HCPs
Enhanced Medical Information portal
Enhancements and additional functionality to Medical Information self-service HCP portal
Add a feature on the Medical Information HCP page to locate their MSL
Medical portal expansion into additional countries
Responses
Infographics
Interactive SRDs
Collapsible/expandable medical answer views
Channels
Expansion of chatbot (3), revisit chatbot
Utilizing new technologies and methods to address HCP questions
Increase accessibility and access to patients and HCPs
Channel expansion
Greater personalization
Text messaging
Social media page
Omnichannel/multichannel delivery
Voicebot
Revisit streamlining dissemination of content to HCPs (text, links, etc.)
Automation and AI Technology
Utilizing AI to address HCP question
AI/ML search
Automated AE/PC detection
Expand insights generation with AI/NLP (2)
Automated translation
AI/NLP
Other technology
Structured/component authoring (2)
Explore letter authoring tools

Multiple mentions indicated in brackets

*CRM* customer relationship management, *HCP* health care professional, *SRD* scientific response document, *AI* artificial intelligence, *ML* machine learning, *AE* adverse event, *PC* product complaint, *NLP* natural language processing

## Discussion

This benchmarking survey of the 32 companies that are part of phactMI™ provided valuable information on the technology and website functionalities of their Medical Information Departments. The main driver for implementing new technology was to remain up-to-date and was not COVID-19 related. One of the barriers that is commonly mentioned for not implementing new technology is the lack of human resources. However, in this survey, 54% of companies that implemented new technology stated that it did not impact personnel resources. This may be an issue of identifying resources through Medical Information versus IT centers.

While there was an increase in the use of chatbots, only 25% of companies are using one. However, 13% are planning on launching one within 12 months and 42% are planning but not sure when. There are still a few companies who do not have plans to implement a chatbot, due to concerns such as cost, Regulatory, Legal, and Compliance concerns, as well as the oversight needed, and time and technology barriers. As the applications of chatbots continue to evolve and gain expanded use, the opportunity to utilize AI generative technology like ChatGPT will become more attractive. As seen a recently published study where chatbot responses to patient questions were rated higher in quality and empathy than physician responses, the use of ChatGPT will significantly impact the opportunities for using AI technology in a chat feature [7].

Social media continues to play a role in providing medical information to customers; therefore, the need to find accurate and evidence-based scientific information remains a key priority. Regardless of the social media audience, HCPs or a patient/caregiver, they can be confident to receive scientifically balanced social media content and response when consuming medical information generated by a pharma organization. Understanding the fine line between promotional versus non-promotional content for each company is important and will continue to play a role in ensuring the type of information posted. For instance, there is a vast difference between organizations with regards to their risk tolerance when posting product information in the form of publications on social media platforms.

While HCP Medical Information websites are so predominant, very few companies have a Medical Information website targeted for patients or caregivers. This is a developing and emerging area across the pharma industry.

As reported through this survey, it is unfortunate that most branded sites do not contain a link to the Medical Information website. This may be due to internal organizational compliance issues of perception of solicitation; however, there may be an opportunity to expand the awareness of Medical Information websites through hyperlinks from other sources of medical information. As HCP websites for medical information are ubiquitous, the content formats are becoming more innovative as demonstrated in this survey.

In assessing how individuals typically find Medical Information websites, 97% of companies’ Medical Information sites are discoverable by search engines including Google™. Another change noted in this survey compared to 2018 was the increase in self-authentication of an HCP to be able to access information on the site. This is in line with the recommendations in the framework for digital provision of medical information [4]. As omnichannel engagement continues to be the new trend, it will be interesting to watch this space for potential content personalization shifts such as registration for specific information or the ability to opt in for updates in the learners’ areas of interest.

While most Medical Information websites allow for searching, the number of documents that are returned can vary from 1–3 to no limit. The rationale concerning applying limits to a search may be a technological limitation or a risk tolerance limitation of the company. A concern with higher numbers of results or no limits is that the results may contain responses that are not specific to the question being asked. The use of keywords may create a self-imposed limit, ensuring the returned documents address the question at hand. However, this too may have limitations resulting in too few documents being returned. One example of keyword tagging for a document concerning ulcerative colitis treatment may be “UC”, “ulcerative”, and “colitis”. With these keywords, any of the three would return the document; however, the term “ulcerative colitis” would not return the document if that string is not a tagged keyword.

Understanding user satisfaction is still critically important to creating impactful and personalized content. A noteworthy observation was that 47% of companies conduct customer satisfaction surveys on their Medical Information websites. This may be primarily because of the less than 1% response rate to such surveys on either HCP or patient sites. It is understandable why companies may not want to dedicate the time and resources necessary for this assessment.

Most companies use multiple technology platforms to perform the core Medical Information functions of CRM, content storage, workflow management, and fulfillment. Most of these platforms can be used for all four functions; however, they are not being utilized to their fullest extent. Only two companies use a single platform for all four functions, highlighting the opportunity for streamlining and integration. Platforms that offer an integrated approach for all four functions would be considered optimal, simplified solutions for Medical Affairs teams.

### Limitations

The direct comparison of the 2018 to 2022 data is limited by many factors. The questions, while attempting to be identical, did have some wording changes and/or additional response options. Additionally, while all 32 companies participated, interpretation of questions may have differed due to the subjective nature of this type of survey and none of the questions were required so some were skipped.

## Conclusion

By engaging the 32 member companies of the phactMI™ network, we have been able to determine how digital innovations are changing the way in which pharmaceutical companies provide Medical Information. Additionally, technological advancements have impacted overall usage of Medical Information search tools and content downloads. The drivers for the implementation of newer technologies and the barriers to implementing newer technologies were also assessed. These survey results allow for the assessment of the current landscape and provide insight into how pharmaceutical companies adapt to keep up with rapidly evolving technological advancements.

Digital engagement that comprises various options such as websites, technologies, applications, social media platforms, or other tools and resources is a subject of interest in the Medical Affairs world to meet technological advances and customer expectations. Consequently, Medical Information content and delivery has evolved significantly over the last decade. It is imperative that Medical Information professionals are aware of and trained on the process as well as implications of implementing new technology. They should become well versed in several key considerations such as their company’s risk tolerance and guardrails and ensure application of internal policies and guidance.

The recommendations from the previous benchmarking survey included developing additional services for patients, continued development of innovative formats of delivery of information, exploring additional website functionalities, improving search engine optimization (SEO), and exploring innovative presentation of information. Based on the results of this survey, SEO, innovative formats of delivery, and website functionalities have been improved with plans for further improvements. Innovative presentation of information, such as apps, are still being evaluated; however, the previous concern around website responsive design is no longer on the forefront of concern as companies have fully embraced responsive design to their sites. Providing patients and caregivers with access to information from Medical Information has progressed slower than other initiatives; however, 30% of companies do now have a patient Medical Information site.

As technology continues to revolutionize and the need to facilitate personalized medical omnichannel content remain a key objective, Medical Information professionals will need to remain agile and transformative in their strategic and tactical planning and execution. These benchmarking survey results provide important insights and guidance on best practices for Medical Information professionals.

Based on this benchmarking survey, the authors recommend that Medical Information Departments focus on the following areas:

1. Continued expansion of services for patients and caregivers to access Medical Information.
2. Deliver omnichannel engagement by exploring opportunities to leverage digital innovations, such as utilizing advanced AI technologies and chatbots, to personalized services offerings and ultimately better engage HCPs and patients/caregivers.
3. Expand the awareness of Medical Information websites through hyperlinks from other sources of medical information and company resources.
4. Streamline and incorporate technology platforms that offer an integrated approach to build efficiencies in workflow of Medical Information Departments.
5. Continuous improvement of all Medical Information solutions to incorporate innovative technologies.

### Supplementary Information

Below is the link to the electronic supplementary material.

Supplementary file1 (DOCX 49 KB)
