# Supplementary material for: An Update from the Benchmark Survey of phactMI™ Member Companies on Providing Medical Information in the Digital Space
Source: Ther Innov Regul Sci. 2023 Nov 10;58(1):200–13. doi: 10.1007/s43441-023-00587-1 (PMC10764438; doi:10.1007/s43441-023-00587-1)
Supplement: Supplementary file 1 — Supplementary file1 (DOCX 49 KB) [file 43441_2023_587_MOESM1_ESM.docx]

Dear phactMI Member:

Thank you for your willingness to participate in our Technology/Website Benchmark Survey. All responses will be kept confidential and only a de-identified, aggregate summary of the data will be shared.

For this survey we will be asking a number of questions that will require data such as metrics of usage/inquiry volume, etc. There are a maximum of 79 questions; however, we have employed skip logic in the design of the survey to eliminate questions that would not be relevant given earlier responses. Your responses will be saved and you will be able to return to the survey if you cannot complete in one session. You will have one month (30 days) to complete the survey.

The sections of the survey include:

- Demographics (up to 9 questions)
- Chatbot (up to 10 questions)
- Social media (up to 4 questions)
- Instant messaging apps (up to 4 questions)
- Websites - Features and functionality (up to 16 questions)
- Websites - Customer satisfaction (up to 5 questions)
- Websites - Discoverable (up to 6 questions)
- Websites - Search function (up to 4 questions)
- Websites - Metrics (up to 8 questions)
- Websites - General (up to 3 questions)
- Technology solutions (up to 10 questions)

Demographics

1. Please provide your Name and company (for tracking purposes only, all data will be deidentified prior to analysis and presentation)
2. How many products is your medical information team responsible for?
   1. < 5 products
   2. 5 - 10 products
   3. 11 - 25 products
   4. 26 - 50 products
   5. >50 products
3. How many of those products were approved in the last 12 months?
4. Is your Medical Information Department global or US only?
   1. Global
   2. US only
   3. Both
5. What percentage of your US medical info inquiries (HCP plus consumer) that you had across your company's portfolio during 2021 (Jan-Dec) are from these channels? (must add to 100%; NOTE: we are trying assess general distribution and understand these will be estimates)
   1. Call center
   2. Chatbot
   3. Website - self-serve
   4. Website - live chat
   5. Dedicated Medical Information app
   6. Social Media - Corporate page(s)
   7. Social Media - Medical Information page(s)
   8. Instant Messaging Apps
   9. Other channel (please identify and provide percent of inquiries)
6. Have you implemented new technology within your medical information department since 2019?
   1. Yes [go to question 7]
   2. No [go to question 9]
7. What new technology has been implemented since 2019? (select all that apply)
   1. Structured Authoring
   2. Scripted Chatbot
   3. AI Driven Chatbot
   4. System Integration (please explain)
   5. Content innovations (i.e., other forms of content delivery such as HTML5, infographics, etc.) (please explain)
   6. Other (please specify)
8. What were the drivers for the implementation of these technologies (select all that apply)?
   1. COVID
   2. Customer expectations
   3. To remain up to date with industry changes
   4. Internal drivers
   5. Omni-channel engagement strategy
   6. Other (please specify)
9. How has technology impacted personnel resources? (open text)

**Chatbot questions**

1. Do you have a chatbot for Medical Information Services?
   1. Yes [go to question 11]
   2. No [go to question 19]
2. When was your chatbot implemented?
3. What was the anticipated chatbot traffic (percent of overall inquiry amount)?
4. What type of chatbot do you have? (Select all that apply)
   1. Free text AI driven bot [go to question 14]
   2. Button based bot (user selects from list of questions/topics) [go to question 15]
   3. Hybrid [go to question 15]
   4. Voice driven (Alexa, google home, etc.) [go to question 15]
   5. Other (please specify) [go to question 15]
5. How well do you feel that the NLP/AI interprets the free text and gets the HCP to the right information?
   1. Most of the time
   2. About 50% of the time
   3. Sometimes
   4. Rarely
6. What type of information does the chatbot provide (select all that apply)?
   1. Short Q&A
   2. Standard Response Documents (SRDs)
   3. Publications
   4. Links to website
   5. Package insert labeling
   6. Technical information only (fit for use, temp excursion, etc.)
   7. Other (please describe)
7. What technology and/or vendor(s) are you using for the chatbot? Free text
8. Have you been able to free-up or redistribute contact center agent time due to the use of your chatbot?
   1. Yes, please specify percentage of time freed-up or redistributed
   2. No
9. Are there any restrictions or limitations with chatbots? (select all that apply) [go to question 20]
   1. No limitations
   2. Yes, on label only
   3. Yes, only certain products covered
   4. Yes, only certain questions covered
   5. Yes, please explain
10. Since you do not currently have a chatbot, are you considering a chatbot? (select all that apply)
    1. Yes, with in 6 months
    2. Yes, with in 12 months
    3. Yes, time unknown
    4. No, cost
    5. No, time
    6. No, compliance issues, legal, or regulatory - please specify concern
    7. No, Technology issues
    8. No, previous experience
    9. No, other - please specify

**Social media**

1. Is your MI staff involved in answering unsolicited medical requests through your corporate social media page? Select all that apply
   1. No
   2. Yes, Facebook
   3. Yes, Twitter
   4. Yes, LinkedIn
   5. Yes, Instagram
   6. Yes, Tik Tok
   7. Yes, other (please specify)
2. Does your medical information department have their own social media page? Select all that apply
   1. No [go to question 24]
   2. Facebook
   3. Twitter
   4. LinkedIn
   5. Instagram
   6. Tik tok
   7. Other (please specify)
3. How many followers do you have on your Medical Information Department’s social media page?

| Feed answer from 21 | <1000 | 1001 - 3000 | 3001 - 5000 | Over 5000 |
| --- | --- | --- | --- | --- |

1. How frequently do you post to your Medical Information Department’s social media page? Feed answer from 21

**Instant messaging app - assuming real time interaction**

1. Which instant messaging application does your Medical Information Department use for external interactions? (Select all that apply)
   1. Do no use any instant message app [go to question 28]
   2. WeChat
   3. WhatsApp
   4. Other, please specify
2. Which countries or regions is the instant messaging application being used? Feed answers from 24
3. Who is responding to these messages?
   1. Vendor (call center)
   2. Artificial Intelligence (AI)
   3. In-house personnel
4. What is the percent of overall inquiries received annually on these channels in non-US countries?

**Website features and functionality**

1. Do you provide medical information through a company Medical Information website?
   1. Yes [go to question 29]
   2. No [go to question 70]
2. Was a commercial platform purchased to support the website or was the website custom developed?
   1. Commercial platform purchased (please specify platform)
   2. Website custom developed (please specify by whom)
3. What web content management system do you use? (Select all that apply)
   1. Drupal
   2. Contentful
   3. Wordpress
   4. Adobe Experience Manager
   5. Other (please specify)
4. Is your Medical Information Website maintained internally or through a vendor? (select all that apply)
   1. Internally, please specify which department is responsible for maintenance
   2. Vendor maintained, please specify vendor
5. Is there a link to the Medical Information Website from your branded websites?

|  | HCP Website | Patient website |
| --- | --- | --- |
| 1. Yes, for all |  |  |
| 1. Yes for some, but not all |  |  |
| 1. No |  |  |

1. Is your Medical Information Website part of a broader Medical Affairs Website
   1. Yes
   2. No

*Website features - HCPs*

1. Do you have a website for HCP's?
   1. Yes [go to question 35]
   2. No [go to question 40]
2. Does your company’s HCP site(s) use any of the following user authentication methods? (select all that apply)

|  | US Based Website | Non-US Based Website |
| --- | --- | --- |
| 1. Self-authentication (HCP clicks a box that says s/he is an HCP) |  |  |
| 1. Validation (User provides Name and NPI number or some other identifying piece of information) |  |  |
| 1. Full registration (User provides multiple pieces of required information) |  |  |
| 1. Register to view specific content (please explain below) |  |  |
| 1. None |  |  |
| 1. Other (please explain) |  |  |

1. Which of the following functionality is available on your HCP website(s)? (Select all that apply)
   1. Live chat (click to chat)
   2. Video chat
   3. Chatbot
   4. Web form for unsolicited request
   5. Web form for AE/PC
   6. 1-800 number
   7. Identify local representative
   8. Identify/request field medical support (MSL)
   9. Other (please specify)
2. What type of information/content format is available on your company’s HCP Medical Information website and how can these content types be accessed/stored? Select all that apply

|  | Search | Navigate to | View | Download | Share | Bookmark/ store |
| --- | --- | --- | --- | --- | --- | --- |
| 1. SRD - pdf |  |  |  |  |  |  |
| 1. SRD - html |  |  |  |  |  |  |
| 1. Slide deck (powerpoint, etc.) |  |  |  |  |  |  |
| 1. Video |  |  |  |  |  |  |
| 1. Webinar |  |  |  |  |  |  |
| 1. Podcast |  |  |  |  |  |  |
| 1. Infographic as response |  |  |  |  |  |  |
| 1. FAQs |  |  |  |  |  |  |
| 1. Link to Clinicaltrials.gov |  |  |  |  |  |  |
| 1. Interactive tools (i.e., ingredients, allergen, temperature check) |  |  |  |  |  |  |
| 1. Publications (i.e., abstracts, manuscripts, journal articles, etc.) |  |  |  |  |  |  |
| 1. Posters |  |  |  |  |  |  |
| 1. Congress presentations compilations |  |  |  |  |  |  |
| 1. AMCP Dossier/ Compendia |  |  |  |  |  |  |
| 1. Investigational product |  |  |  |  |  |  |
| 1. Medical education materials |  |  |  |  |  |  |
| 1. disease state material |  |  |  |  |  |  |
| 1. shared marketing material |  |  |  |  |  |  |
| 1. Other (please explain) |  |  |  |  |  |  |

1. If you have SRDs in HTML format, how did you develop the content?
   1. We do not have SRDs in HTML format
   2. Outside agency
   3. Internal developers
   4. Automated process (please explain)
2. Which of the following content type(s) on your HCP website goes through Medical, Legal, Regulatory, Compliance and/or other function review? (Select all that apply)
   1. Website framework prior to launch
   2. Content available without searching (navigate to)
   3. Scientific response documents
   4. New features
   5. Chatbot
   6. Others (please specify)

*Website features - patients/caregivers*

1. Do you have a website for patients/caregivers?
   1. Yes [go to question 41]
   2. No [go to question 44]
2. Which of the following functionality is available on your patient/caregiver website(s)? (Select all that apply)
   1. Live chat
   2. Video chat
   3. Chatbot
   4. Web form for unsolicited request
   5. Web form for AE/PC
   6. 1-800 number
   7. Other (please specify)
3. What type of information/content format is available on your company’s Patient/Consumer Medical Information website and how is it accessed?

|  | View | Download | Store on site | Share | Bookmark |
| --- | --- | --- | --- | --- | --- |
| 1. SRD - pdf |  |  |  |  |  |
| 1. SRD - html |  |  |  |  |  |
| 1. Slide deck (PowerPoint, etc.) |  |  |  |  |  |
| 1. Video |  |  |  |  |  |
| 1. Webinar |  |  |  |  |  |
| 1. Podcast |  |  |  |  |  |
| 1. Infographic as response |  |  |  |  |  |
| 1. FAQs |  |  |  |  |  |
| 1. Link to Clinicaltrials.gov |  |  |  |  |  |
| 1. Interactive tools (i.e., ingredients, allergen, temperature check) |  |  |  |  |  |
| 1. Publications (i.e., articles, posters, congress materials, etc.) |  |  |  |  |  |
| 1. AMCP Dossier/ Compendia |  |  |  |  |  |
| 1. Patient education materials (Commercial created) |  |  |  |  |  |
| 1. Patient education materials (Medical created) |  |  |  |  |  |
| 1. Other (please explain) |  |  |  |  |  |

1. Which of the following content type(s) on your patient/caregiver website goes through Medical, Legal, Regulatory, Compliance and/or other function review? Select all that apply
   1. Website framework prior to launch
   2. Content available without searching
   3. Scientific response documents
   4. New features
   5. Chatbot
   6. Other (please specify)

**Website customer satisfaction**

1. Do you have a customer satisfaction survey on your website?
   1. Yes [go to question 45]
   2. No [Go to question 49]
2. How are you serving up the customer satisfaction survey?

|  | HCP site | Patient site |
| --- | --- | --- |
| - 1. In the beginning of interaction |  |  |
| - 1. Pop-up during the interaction |  |  |
| - 1. At the end of the interaction |  |  |
| - 1. Other (please specify) |  |  |

1. Do have an approximate response rate
   1. Yes, patient website please provide approximate rate
   2. Yes, HCP website, please provide approximate rate
   3. No
2. What are you asking about in your customer satisfaction survey? (select all that apply)
   1. Content: quality and level of information
   2. Content: format (i.e., traditional SRD, navigable SRD, pdf, infographics, etc.)
   3. Overall Experience
   4. Customer effort (i.e, how easy was it get the information)
   5. Net promoter (i.e., would you recommend this site to a friend/colleague)
   6. Value of information to support patient care/clinical decision
   7. Others (please specify)
3. What is the format of the questions in the customer satisfaction survey? (select all that apply)
   1. Likert scale
   2. Multiple choice
   3. Select all that apply
   4. Yes/No
   5. Open ended

**Website discoverable**

1. Is your medical information/medical affairs site discoverable on Google and/or other search engines?
   1. Yes
   2. No
2. Are your answers/content directly discoverable on Google and/or other search engines?

|  | On-label information | Consistent with label information | Off-label information |
| --- | --- | --- | --- |
| - 1. Yes |  |  |  |
| - 1. No |  |  |  |

1. Are you tagging content in your website?
2. Yes (please explain what is tagged)
3. No
4. Do you have resources dedicated to Search Engine Optimization (SEO) for your Medical Information Website?
   1. Yes [go to question 53]
   2. No [go to question 55]
5. What group(s) and resources are utilized for SEO? (free text)
6. Are you using Search Engine Marketing (paid search) to facilitate customers finding your content?
   1. Yes, please provide details
   2. No

**Website search function**

1. Which of your MI department websites have a search function? (select all that apply)
   1. None of the MI department websites have a search function [go to question 59]
   2. Healthcare provider site has a search function [go to question 56]
   3. Patient/caregiver site has a search function [go to question 56]
2. What type of search functionality do you use on the website? (select all that apply)
   1. Key words - looks for matching documents that contain one or more of the key words specified
   2. Full text (also called Free text) - Looks for documents that contain the searched words in addition to their synonyms and metadata
   3. AI/Natural language processing - Matching documents are surfaced when users types/speaks words in everyday language as opposed to just key words
   4. Drop down lists - products
   5. Drop down lists - topics
   6. Other (please explain)
3. What is the limit for the number of results returned?
   1. 1 - 3 results
   2. 4 - 5 results
   3. 6 - 10 results
   4. Over 10 results
   5. No limit on results (please explain rationale)
4. What information formats are returned in the search? (select all that apply)
   1. SRD - pdf
   2. SRD - html
   3. Slide deck (PowerPoint, etc.)
   4. Video
   5. Webinar
   6. Podcast
   7. Infographic as response
   8. FAQs
   9. Website Link to Clinicaltrials.gov
   10. Interactive tool (ie ingredient checker, allergen checker, temperature checker)
   11. Publications (ie abstracts, manuscripts, journal articles,)
   12. Posters
   13. Congress materials (Congress presentations, compilations)
   14. Educational materials
   15. Other (please explain)

**Metrics**

1. Do you capture metrics for your medical information websites?
   1. No metrics captured [go to question 67]
   2. Yes [go to question 60]
2. What tools are used for tracking website usage metrics?
   1. Adobe analytics
   2. Google analytics
   3. Web trends
   4. Other (please specify)
3. What is the traffic on your Medical Information channel in terms of visitors per year?
   1. HCP Website __________
   2. Patient Website _________
4. What is the traffic on your Medical Information Website in terms of number of page views per year? (free text)
5. What is the frequency of same visitors? (free text)
6. What is the number of content downloads per year? (free text).
7. What is the average time (in minutes) visitors spend on the Medical Information Website? (free text)
8. List how customers are coming to your website (percentage should equal 100%)
   1. Google
   2. Bing
   3. Yahoo
   4. Other search engine (please specify)
   5. Brand website
   6. Corporate website
   7. Social media
   8. Other (please specify)
   9. Unsure

**Website - general**

1. What methods are used to inform HCPs of your websites (select all that apply)
   1. None
   2. Field Medical - verbally
   3. Field Medical - business card or other material
   4. Field Sales - verbally
   5. Field Sales - business card or other material
   6. Email
   7. Banner ads
   8. Link on Medical Information letters/correspondence
   9. Social media
   10. Other (please specify)
2. What methods are used to inform patients of your website(s) (select all that apply)
   1. Do not have a patient website
   2. Do not inform patients of our website
   3. Link on patient medical information letters or correspondence
   4. Banner ads
   5. Email
   6. Social media
   7. Other (please specify)
3. What are you doing to broaden the reach of your website?
   1. Electronic medical record (please specify provider)
   2. Partnering with third party sources (ie Medscape, Sermo, Epocrates, etc. ) (please specify)
   3. Social media
   4. Other please specify

**Technology solutions**

1. How are you communicating medical information trends to internal stakeholders? (select all that apply)
   1. Word clouds
   2. Insights
   3. Newsletter
   4. Email
   5. In person/Virtual Meeting
   6. Other
2. Describe the technology/system you use for insights and analytics of inquiries including reports or dashboards? (select all that apply)
   1. Spotfire
   2. Tableau
   3. Geodart
   4. Qlik sense
   5. Other (please specify)
3. Who performs the analytics on inquiry data?
   1. Vendor
   2. IT Staff
   3. MI Staff
   4. MI Staff with specific skills (please specify specific skills)
4. Do you use a vendor to answer unsolicited medical information requests? (Select all that apply)
   1. No
   2. Yes, please specify vendor
5. Do you use any of the following technologies?
   1. Speech to text technology (please specify which technology)
   2. Automated translation services (please specify which technology)
   3. No
6. What tools/format do you use for your content creation?
   1. Abortext
   2. Authorit
   3. IRMS
   4. Microsoft PowerPoint
   5. Microsoft Word
   6. Veeva
   7. Other (please specify)
7. Which of following technology platforms do you use for the various functions (select all that apply)?

|  | Customer Relationship Management for inquiries (not MSL System) | Content Storage | Content workflow management system | Fulfillment/ package creation system |
| --- | --- | --- | --- | --- |
| None |  |  |  |  |
| Documentum |  |  |  |  |
| Docuvera |  |  |  |  |
| IRMS |  |  |  |  |
| Mavens |  |  |  |  |
| Med inquirer |  |  |  |  |
| Salesforce |  |  |  |  |
| Sharepoint |  |  |  |  |
| Siebel |  |  |  |  |
| Veeva |  |  |  |  |
| Other (please specify) |  |  |  |  |
|  |  |  |  |  |

1. Are your technology platforms global, regional, or local?

|  | Global | Regional | Local |
| --- | --- | --- | --- |
| - - - - 1. Website framework |  |  |  |
| - 1. Inquiry intake |  |  |  |
| - 1. Content creation |  |  |  |
| - 1. Content workflow management |  |  |  |
| - 1. Chatbot |  |  |  |
| - 1. Social media |  |  |  |
| - 1. Other |  |  |  |

1. What other MI-related technologies are you working on? (please elaborate)
2. What is on your 3-year horizon for MI-related technology? (please elaborate)
